# Improving Efficacy and Reducing Systemic Toxicity: An In Vitro Study on the Role of Electrospun Gelatin Nanofiber Membrane for Localized Melanoma Treatment

**DOI:** 10.3390/bioengineering12090910

**Published:** 2025-08-25

**Authors:** Jason Sun, Yi-Chung Lai, Bing-Wu Shee, Chih-Hsiang Fang, Ching-Yun Chen, Jui-Sheng Sun

**Affiliations:** 1 Carmel Catholic High School, One Carmel Parkway, Mundelein, IL 60060, USA; jasonsun112007@gmail.com; 2 Department of Orthopedic Surgery, National Taiwan University Hospital, No. 7, Chung-Shan South Road, Taipei 10002, Taiwan; yclaintuh@ntuh.gov.tw (Y.-C.L.); danny07291991@hotmail.com (C.-H.F.); 3 Department of Orthopedic Surgery, Landseed International Hospital, No. 77, Guangtai Road, Pingzhen District, Taoyuan 324609, Taiwan; sheebw@landseed.com.tw; 4 Department of Biomedical Sciences & Engineering, National Central University, No. 300, Zhongda Rd., Zhongli District, Taoyuan 320317, Taiwan

**Keywords:** melanoma, cisplatin, chemotherapy, drug delivery, gelatin nanomembrane

## Abstract

Malignant melanoma is a highly metastatic skin cancer, representing about 5% of all cancer diagnoses in the United States. Conventional chemotherapy often has limited effectiveness and severe systemic side effects. This study explores a localized, topical delivery system using cisplatin-loaded nanomembranes as a safer and more targeted alternative. Cell viability assays established the safe cisplatin concentrations for tissue culture. Gelatin-based nanomembranes incorporating cisplatin were fabricated via electrospinning. Biocompatibility and therapeutic efficacy were tested by applying the membranes to cultured melanoma and normal skin cells. Controlled drug release profiles were evaluated by adjusting cross-linking times. Cisplatin concentration between 3.125 and 12.5 µg/mL were found safe. Nanomembranes with these doses effectively eliminated melanoma cells with minimal harm to healthy skin cells. Drug-free membranes showed high biocompatibility. Cross-linking duration allowed tunable and stable drug release. Cisplatin-loaded gelatin nanomembranes offer a promising topical therapy for melanoma, enhancing drug targeting while reducing systemic toxicity. This approach may serve as a cost-effective alternative to systemic treatments like immunotherapy. Future research will focus on in vivo testing and clinical application.

## 1. Introduction

Malignant melanoma is recognized as one of the most aggressive and deadly forms of skin cancer. Although it comprises only about 3% of all diagnosed skin cancer cases, it is responsible for approximately 65% of all skin cancer deaths [1]. The increasing incidence of melanoma is closely associated with heightened ultraviolet (UV) exposure from sunlight and tanning beds, especially among the white population in the United States [2,3]. Chronic exposure to ultraviolet radiation (UVR) induces oxidative stress in melanocytes, the pigment-producing cells of the skin, resulting in DNA damage and gene expression dysregulation [4,5]. Over time, the accumulation of these mutations can lead to uncontrolled melanocyte proliferation, tumor formation, and metastasis to distant organs [5]. Without effective preventative measures, global melanoma cases are projected to reach 510,000 by 2040 [6].

Current standard treatments for metastatic melanoma—following wide surgical excision—include immunotherapy, targeted therapy, and immune checkpoint inhibitors [2,7,8,9]. While therapies have significantly improved patient outcomes, they are associated with substantial financial burdens, often amounting to hundreds of thousands per patient [10]. In contrast, chemotherapy, once a mainstay in melanoma treatment, has become less favored due to its limited impact on survival and its broad systemic side effects [7,11]. For instance, cisplatin, a widely used platinum-based chemotherapeutic agent, induces cancer cell death by forming crosslinks with purine bases in DNA, thereby inhibiting repair and triggering apoptosis [7,12]. However, cisplatin lacks selectivity, damaging healthy cells along with cancer cells. Its systemic administration is linked to various complications, including nephrotoxicity, liver dysfunction, peripheral neuropathy, and bone marrow suppression [7,12,13,14]. These limitations underscore the need for localized drug delivery systems that minimize systemic toxicity while maintaining therapeutic efficacy.

Nanotechnology, a rapidly evolving interdisciplinary field, has catalyzed transformative advances in medicine, particularly in diagnostics, tissue engineering, gene therapy, and drug delivery [15,16]. Among its many applications, nanofibers fabricated via electrospinning have emerged as a promising platform for localized drug delivery. Electrospinning utilizes high-voltage electrostatic forces to draw polymer solutions into ultrafine fibers with diameters typically ranging from 50 to 500 nm [17]. In this process, a charged polymer solution forms a Taylor cone at the nozzle tip; as the jet travels through the air, the solvent rapidly evaporates, depositing solid fibers onto a grounded collector [17,18,19]. The resulting nanofibers offer a high surface area-to-volume ratio, porosity, and drug-loading capacity—characteristics that make them ideal for sustained, targeted chemotherapy delivery with lower dosage than traditional systemic administration [17,19,20].

Several studies have highlighted the efficacy of nanofiber-based drug delivery in cancer therapy. For instance, Tseng et al. developed a biodegradable electrospun membrane loaded with carmustine, irinotecan, and cisplatin to treat glioblastoma multiforme, reporting high drug release with minimal brain inflammation in both in vitro and in vivo models [21]. Similarly, Zong et al. (2015) created cisplatin-loaded polylactide nanofibers for cervical cancer, achieving high local drug concentrations with limited systemic toxicity [22]. However, many nanofiber systems reported in research still rely on synthetic polymers, which may be toxic and difficult to scale for mass production [19,23].

To address these challenges, this study aims to develop electrospun gelatin nanomembranes as a localized drug delivery system for melanoma treatment. Gelatin, a natural polymer derived from collagen, was selected for its biocompatibility and structural similarity to the extracellular matrix [18]. The electrospun gelatin fibers will be loaded with cisplatin to provide targeted therapy with minimized systemic exposure. This approach seeks to offer a cost-effective, efficient alternative to existing melanoma treatments, potentially reducing both clinical side effects and the financial burden on patients.

## 2. Materials and Methods

### 2.1. Design Plan

**Figure 1:**
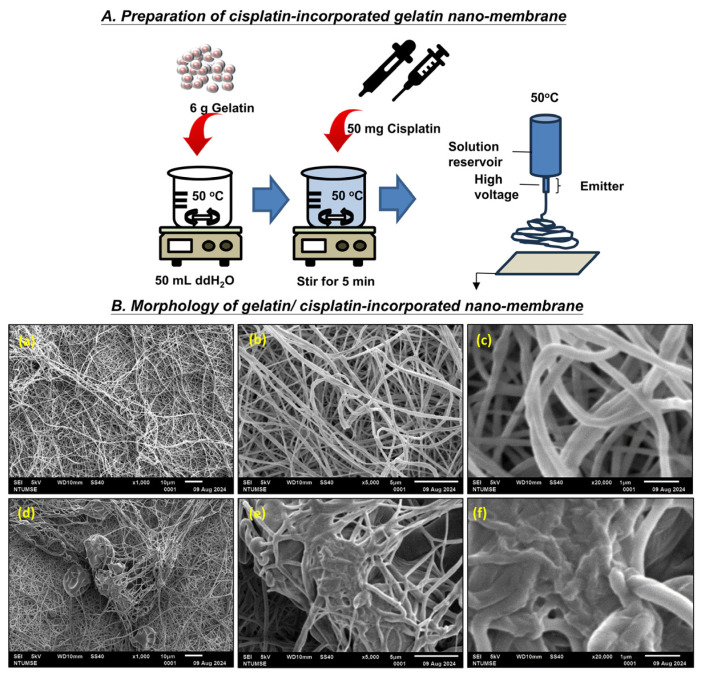
Preparation and morphology of cisplatin-incorporated gelatin nanomembrane. (**A**) Schematic of the electrospinning process used to fabricate cisplatin-loaded gelatin nanofibers. (**B**) Schematic representation of nanofibers. SEM images showing the morphology of the nanomembranes. Smooth, interconnected fibers were observed, with average diameters of 150 ± 50 nm (scale bars = 5 µm). (**a**–**c**) Pure gelatin nanofibers; (**e**–**f**) cisplatin-loaded gelatin nanofibers.

**Scheme 1:**
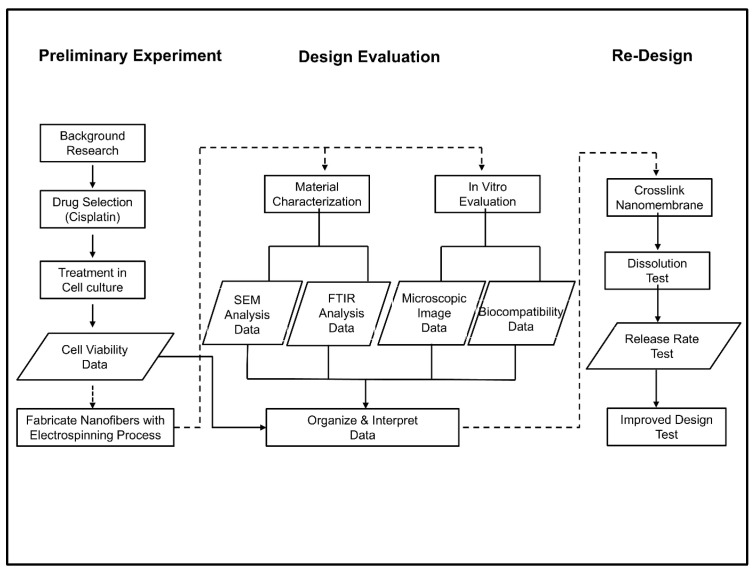
Design process flow chart.

The design process starts with preliminary experimentation to study the cytotoxicity of cisplatin to cell cultures at different concentrations, and the resulting data guide the fabrication of drug-carrying nanomembranes. The nanomembranes are then characterized and evaluated in vitro with cell cultures; then, the nanomembrane is redesigned (Figure 1 and Scheme 1).

### 2.2. Cell Culture

L929 fibroblasts ((ATCC # CCL-1): C3H/An male mouse fibroblast cell line), B16F10 melanoma cells ((ATCC^®^ CRL-6475™): murine melanoma cell line from C57BL/6J mouse), and HaCaT cells (immortalized human keratinocytes) were cultured in DMEM (Gibco by Thermo Fisher Scientific, Waltham, MA, USA) supplemented with 10% (*v*/*v*) fetal bovine serum (FBS, Gibco by Thermo Fisher Scientific), 1% (*v*/*v*) penicillin−streptomycin (Gibco by Thermo Fisher Scientific), and 1% (*v*/*v*) GlutaMAXTM (Gibco by Thermo Fisher Scientific) Supplement. Before each experiment, the cultures were washed with phosphate-buffered saline (PBS, pH 7.4, Gibco by Thermo Fisher Scientific), detached with 0.25% trypsin−ethylenediaminetetraacetic acid (EDTA) solution (Thermo Fisher Scientific), and then centrifuged at 1000 rpm for 5 min, followed by resuspension.

### 2.3. CCK-8 Cell Viability Assay

The MTT assay determined the cytotoxicity, and the Cell Counting Kit-8 (CCK-8) assay measured the cell viability [24,25]. It is also suggested that CCK-8 exhibits higher sensitivity than other toxicity-based tetrazolium assays, such as MTT or XTT; in this study, we selected CCK-8 for the cell viability assay. Briefly, cells were first counted, and 1 × 10^4^ cells (of each respective cell type) per well were seeded in a 96-well cell culture plate (Corning Inc., Corning, NY, USA). Then, after incubation at 37 °C in a humidified atmosphere with 5% CO_2_ for 24 h, the culture medium was treated with different concentrations (100, 50, 25, 12.5, 6.25, and 3.125 µg/mL) of cisplatin. Four replicates were made for each measurement, and multiple experiments were conducted over varying durations (24, 48, and 72 h) to observe the effects of the chemotherapeutic agent over time. Finally, a CCK-8 cell viability assay was performed to analyze the results between the 3 types of tissue culture. Briefly, 10 μL of the CCK-8 reagent (MedChemExpress Ltd., Princeton, NJ, USA) was added to each well, and OD at 450 nm was measured using a multifunction microplate reader (Infinite M200 Pro, Tecan Life Science, Männedorf, Switzerland) after incubation for 2 h at 37 °C. The percentage of each concentration relative to the control was presented as cell viability. The half maximal inhibitory concentration (IC50) value was calculated using SPSS 4.3.

### 2.4. Electrospinning

As the structure and arrangement of collagen bear a strong similarity between human and porcine [26], we chose porcine gelatin as a better candidate for human application in the future. Homogeneous gelatin–cisplatin solutions were prepared at 50 °C by dissolving 6 g of gelatin (Type A, porcine, Sigma-Aldrich, St. Louis, MO, USA) and 50 mL cisplatin (Kemoplat, Cisplatin Injection BP: 1 mg/1 mL, Fresenius KABI, Bad Homburg, Germany) in ddH_2_O solutions with concentrations of 100 µg/mL, 12.5 µg/mL, 6.25 µg/mL, 3.125 µg/mL, and 0 µg/mL using a magnetic stirrer (Figure 1A). The resulting solution was transferred to the syringe of the electrospinning machine (ESM) and propelled through the plastic tube towards the needle outlet (with an inner diameter of 1.5 mm) ejector. A potential of 20 kV, generated by a high-voltage power supply (SC-PME50, Cosmi Global Co., Ltd., New Taipei City, Taiwan), was subsequently applied to the droplets of the discharged solution. The newly formed fibers were eventually collected by a rotating cylindrical aluminum collector 7 cm away from the ejector. To prevent premature gelation of the dissolved gelatin, the internal temperature was maintained between 45 °C and 70 °C by the internal heater (AREX-6; VELP Scientifica, Deer Park, NY, USA) throughout the process.

### 2.5. Scanning Electron Microscopy with Energy Dispersive X-Ray Spectroscopy (SEM-EDX)

The fixed scaffolds were carefully mounted onto aluminum stubs and sputter-coated with gold to enhance surface conductivity and minimize charging effects. The morphological features and elemental compositions were examined using the Hitachi S-4500 field emission scanning electron microscope (Tokyo, Japan). The microscope was operated at an accelerating voltage of 20 kV and a 9.0 mm working distance to ensure high-resolution imaging. SEM provided a visual micrograph of the nanomembrane surface, while the EDX system yielded precise identification and quantification of element distributions.

### 2.6. Fourier-Transform Infrared Spectroscopy (FTIR) Analysis

At the time of spectroscopy (Nicolet iS20, Thermo Fisher, Taipei, Taiwan), vials containing 5 mg of freeze-dried composite fabrics analyzed in this study were ground and mixed thoroughly with 95 mg of potassium bromide. A small amount of this mixed powder was put in the sample holder of a PerkinElmer Spectrum One instrument equipped with a TGF detector and analyzed using the DRIFT (Diffuse Reflectance Infrared Fourier Transform) method. Pure potassium bromide was used as the control for background spectra.

For each spectrum, a total of 3600 scans, at 1 cm^−1^ resolution, were applied in the range of 400–4000 cm^−1^. Spectral mathematical manipulations (such as baseline corrections, smoothing, and deconvolution) and statistical parameters (such as peak frequency shifts and intensity ratios of various infrared bands) were calculated using PerkinElmer software 1B, version 3.02.

### 2.7. Cell Viability with Cisplatin-Incorporated Nanomembrane

L929 fibroblasts, B16F10 melanoma cells, and HaCaT human keratinocytes were cultured and prepared as previously mentioned. Freshly harvested cells (3 × 10^5^ cells of each cell type) were seeded on the 3 cm 6-well plate. After 24 h of incubation, the original media were removed, 1 × 1 cm pieces of nanomembrane (cisplatin-incorporated or non-incorporated) were applied to the cell culture surface, and 3 mL of DMEM growth medium was replenished. Cell cultures were treated over 1 h, 5 h, and 24 h. At the end of each experiment, cell morphologies at the cell-nanomembrane junction were observed under the inverted microscope to assess viability. According to the manufacturing process for nanofiber membrane described above (for 3.125 µg/mL in electrospinning solution), each 1 × 1 cm nanomembrane used in this test contains 0.065 µg of cisplatin, while 1 mL medium contains 3.125 µg of cisplatin (Table 1). ImageJ 1.54 was used to measure cell area in digital images.

### 2.8. Release Rate Studies

Platinum traces can be determined in the form of the PtCl(6)(2-) complex I hydrochloric acid solution by measuring the absorbance at 260 nm; due to the limitation of UV–visible spectrophotometry, ketoprofen was used instead of cisplatin for release profiling in this study. Briefly, ketoprofen was supplied by Sigma-Aldrich (K1751, Saint Louis, MO 63103, USA). The controlled release tests were prepared using electrospinning as described above, with 50 mg ketoprofen instead of 5 mg cisplatin. For the standard curve of ketoprofen, 50 mg of ketoprofen was weighed accurately and dissolved it in 100 mL of methanol to prepare the stock solution. The prepared stock solution was subsequently diluted with 5 mL of 0.05 M phosphate buffer to obtain solution concentrations of 1.563, 3.125, 12.5, 25, and 50 μg/mL. The electro-spun fiber mats were collected, and the as-spun fibers were dried in a vacuum oven at 30 °C for 24 h and then exposed to 1% glutaraldehyde (Sigma-Aldrich, Saint Louis, MO, USA) at room temperature for 0, 12, or 48 h. Fibers for cell culture were immersed in a 0.1 M glycine aqueous solution for 1 h to abate the toxicity of residue aldehyde groups to cells, followed by sterilization by dipping in 70% (*v*/*v*) ethanol (20 times) for drying and overnight UV exposure, and then stored for different treatment conditions.

At each pre-determined time period, a dissolution test was performed on 4 samples. An amount of 100 mL of distilled water was used as the dissolution medium. Samples from the dissolution medium were withdrawn using a syringe at intervals of 1 day, and every day up to 7 days from all 4 vessels for all the formulations. They were then placed separately in labeled test tubes. The absorption of maximum ketoprofen was measured at 260 nm using a UV–Visible Evolution 60 spectrophotometer (Thermo Scientific). A new standard curve was freshly prepared for every dissolution test. The mean absorbance values of the 4 samples were then converted to drug concentrations, and the percentage of drug release was plotted [27]. The blank will be phosphate buffer at pH 7.4. The readings were then recorded.

### 2.9. Statistical Analysis

All quantitative data were expressed as mean value ± standard deviation unless otherwise specified. A two-sample, two-tailed *t*-test was performed on all collected data to compare group means with the control group. Statistical significance was defined as a *p*-value less than 0.05. All analyses were performed using SPSS version 16.0 software.

## 3. Results

### 3.1. Scanning Electron Microscopy–Energy Dispersive X-Ray Spectroscopy (SEM-EDX) Analysis

#### 3.1.1. Morphology

The morphology of the cisplatin-incorporated gelatin nanomembrane is shown in Figure 1B (a–c: gelatin nanomembrane; e–f: cisplatin-incorporated gelatin nanomembrane), which features nanofibers characterized by smooth surface textures, high entanglements, and uniform diameters around 150 ± 50 nm. The nanomembranes exhibited high porosity and adequate interconnectivity, while the presence of cisplatin in low concentrations caused no significant difference in morphological properties at the microscopic level.

#### 3.1.2. Characterization of Materials

**Figure 2:**
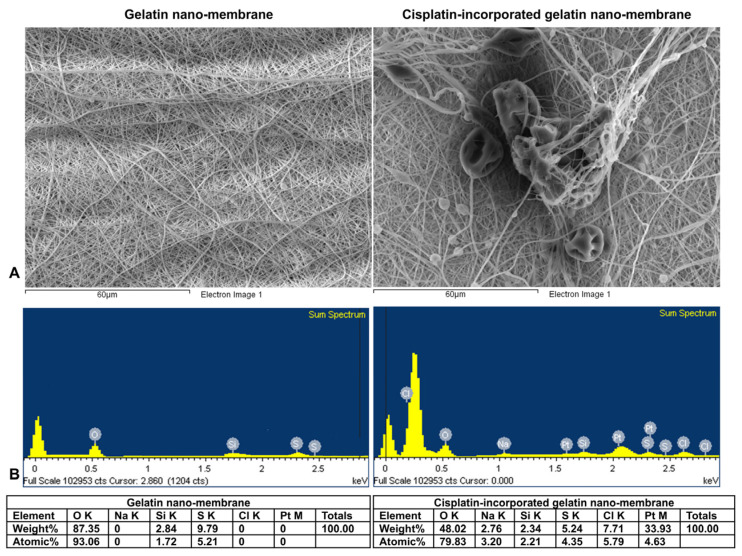
SEM-EDS photomicrographs showing cisplatin crust on the nanofibers’ surface. (**A**) SEM-EDS mapping images of the nanofiber surface. (**B**) EDS spectra of 100 µg/mL cisplatin-incorporated nanomembrane. Processing option: all elements were analyzed and normalized. **Note:** the electron shells (e.g., K, L, M) correspond to the respective X-ray emission origins. N = 4 scans.

Cisplatin [cis-diamminedichloroplatinum(II)], cis-[Pt(NH_3_)_2_Cl_2_], is a square planar coordination complex with two ammonia (NH_3_) molecules and two chloride (Cl^−^) ions acting as ligands. The scanning electron microscopy–energy dispersive X-ray spectroscopy (SEM-EDX) spectra presented in Figure 2 confirmed the successful incorporation of cisplatin into the gelatin nanofibers by detecting the characteristic signals of platinum and chloride elements in the sample. The results of the microanalysis showed an increase in the Cl atomic ratio of 1:5.97 (pre-gelatin: cisplatin-incorporated gelatin nanomembrane), providing a good demonstration of the cisplatin particles’ incorporation into the nanofibers.

### 3.2. Fourier-Transform Infrared Spectroscopy (FTIR) Analysis

**Figure 3:**
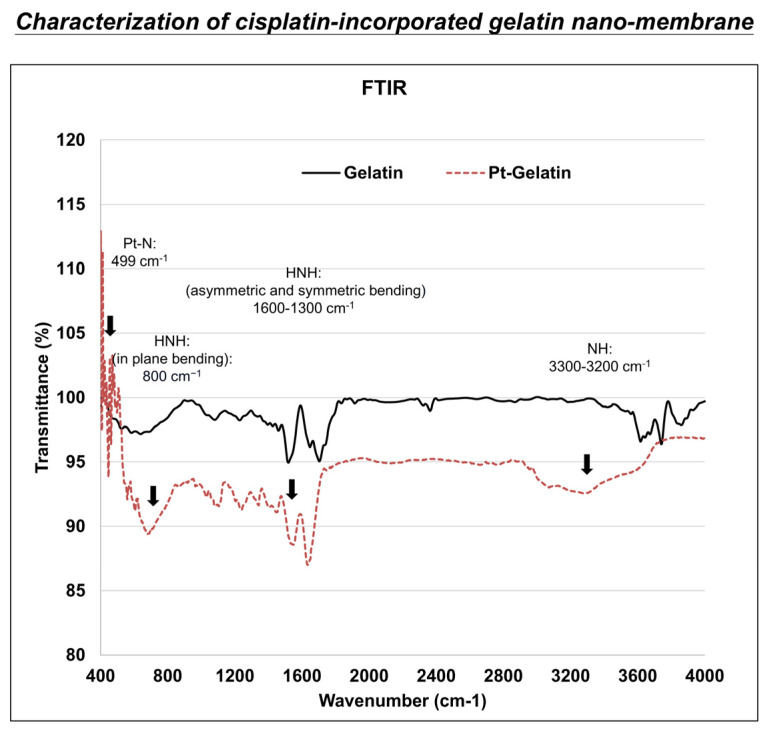
FTIR characterization of cisplatin-incorporated gelatin nanofibers, showing key spectral shifts indicative of drug–polymer interactions. Characteristic spectral changes were observed, particularly in the following regions: 3300–3200 cm^−1^ (asymmetric and symmetric stretching of NH groups), 1600–1300 cm^−1^ (asymmetric and symmetric bending of H-NH) region, around 800 cm^−1^ (H-NH in-plane bending), and 499 cm^−1^ (Pt-N stretching vibration).

Figure 3 shows the FT-IR spectra of the cisplatin-incorporated gelatin nanofiber. As is evident in Figure 3, the FT-IR spectrum of the sample after the addition of Cis-Pt is reported. Changes are expected in the 3300–3200 cm^−1^ region (related to asymmetric and symmetric stretching of NH groups), the 1600–1300 cm^−1^ region (related to HNH asymmetric and symmetric bending), and around 800 cm^−1^ (related to HNH in-plane bending and stretching vibration of Pt-N (499 cm^−1^) [28,29].

#### Cell Viability Assay

**Figure 4:**
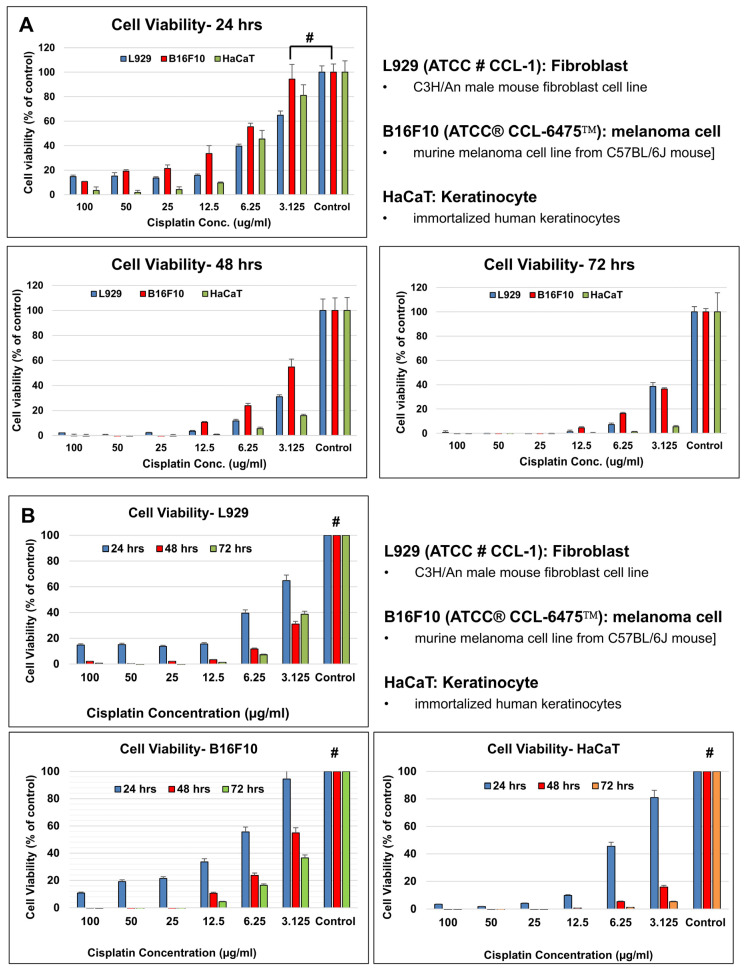
Cell viability after treatment with varying cisplatin concentrations, measured by CCK-8 assay. Cell viability decreases with cisplatin concentration (**A**) and increased incubation time (**B**). n = 4; #: *p* > 0.05.

The cell viability was examined using the CCK-8 assay (Figure 4). Our results showed that all cell types have better cell viability at the lower cisplatin concentrations, while the cell viability of B16F10 (melanoma) cells seem to have a higher viability than those of L929 (fibroblast) cells and HaCaT (keratinocyte) cells. The cell viability decreased as the period of cisplatin incubation time increased. At 24 hours of cisplatin incubation, the cell viability of all three cell types was still higher than 60% in the group containing 3.125 µg/mL of cisplatin. The viability of the B16F10 (ATCC^®^ CCL-6475™) melanoma cell line was similar to that of the control samples (Figure 4; #: *p* > 0.05; Table 2). We chose this concentration for further cell viability studies with the cisplatin-incorporated nanomembrane.

Our results showed that all cell types had better cell viability at lower cisplatin concentrations, while the cell viability of B16F10 (melanoma) cells appeared higher than that of L929 (fibroblast) cells and HaCaT (keratinocyte) cells. The cell viability decreased as the period of cisplatin incubation increased; at 24 hours of cisplatin incubation, the cell viability of all three cell types was still higher than 60% (n = 4; #: *p* > 0.05).

### 3.3. In Vitro Evaluation of Nanomembranes

#### 3.3.1. Material Biocompatibility

As shown in Table 3, all experimental groups (excluding the positive control) resulted in cell viabilities greater than 70%, thereby meeting the standards of ISO 10993-5 for in vitro cytotoxicity. In fact, the addition of pure (0 µg/mL) gelatin nanomembranes in DMEM further enhanced cell growth. Experimental groups with 100%, 75%, and 50% of the test solution all exhibited higher cell viabilities compared to the negative control. This enhancement in cell viability is likely attributed to gelatin’s ability to mimic the extracellular matrix and provide a structure for tissue regeneration [18].

#### 3.3.2. Cell Viability with Cisplatin-Incorporated Nanomembrane: 2D Qualitative Study

**Figure 5:**
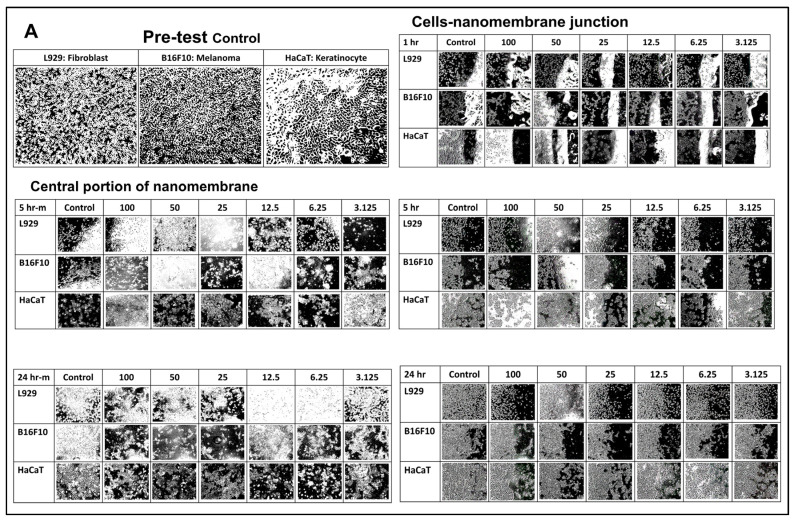

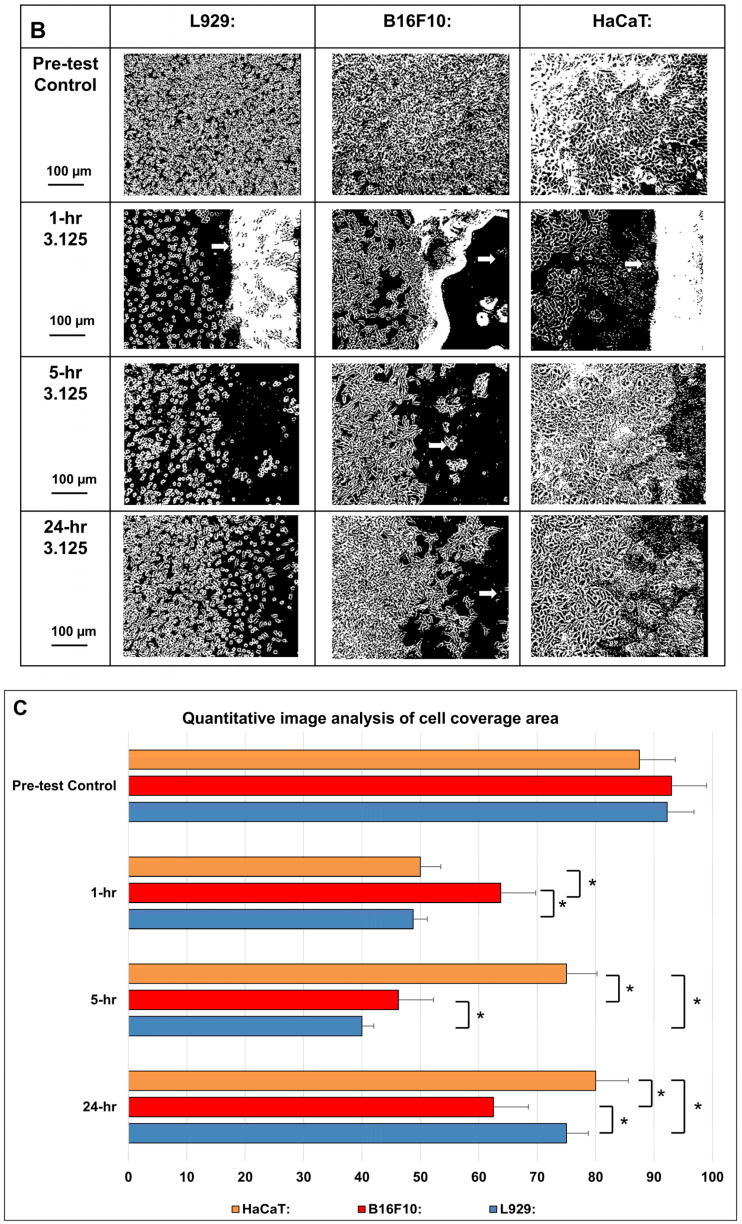
Cell–nanomembrane interaction at 1 h, 5 h, and 24 h post-application. (**A**) Low magnification: whole membrane area and junction. (**B**) High magnification: junction region at 3.125 µg/mL concentration. Note: pre-test control = no membrane or cisplatin; control = gelatin membrane only; white arrows (=>): shrinkage of cells. (**C**) Quantitative image analysis of cell coverage area. (*: *p*< 0.05).

The local interaction between different cell types and cisplatin-incorporated nanomembrane were demonstrated at Figure 5. At 1 h after nanomembrane application, shrinkage of cells was observed in all cell populations near the junction, while the cells within the coverage of the nanomembrane showed severe shrinkage, with some cell debris visible. At 5 h after nanomembrane application, shrinkage of cells was still observed in cell populations near the junction for the control samples, while the recovery of cell populations within the coverage of the nanomembrane showed some improvement, especially for the L929 and HaCaT cells. At 24 h after cisplatin-incorporated nanomembrane application, the shrinkage of cells kept improving in cell populations near the junction, while the recovery of cell populations within the coverage of the nanomembrane showed nearly complete recovery for the control samples. The cytotoxic effect was more obvious in the B16F10 cells, especially at the highest concentration, whereas at the lower concentrations (6.25/3.125), the cytotoxic effect on L929 and HaCaT cells seemed less obvious (Figure 5A,B).

#### 3.3.3. In Vitro Dissolution Study

**Figure 6:**
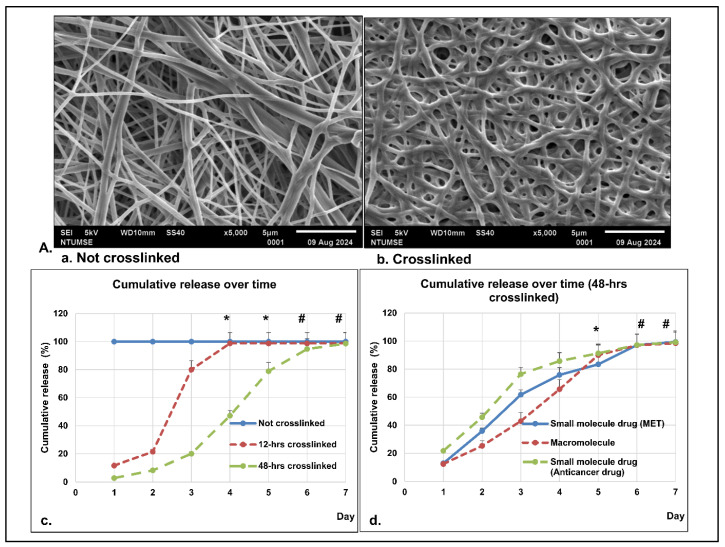

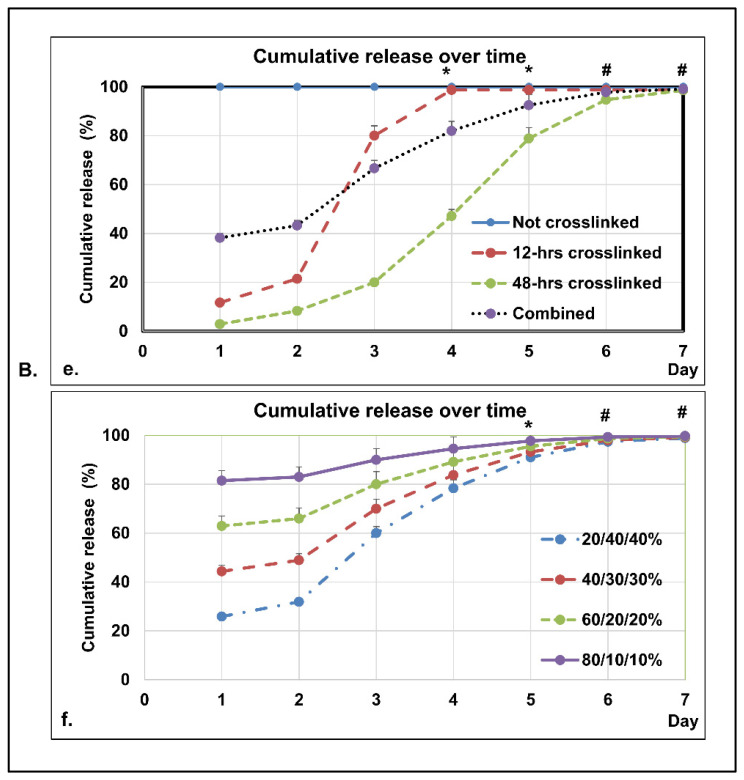
Cumulative release profile of ketoprofen and different molecules with/without cross-linking, and a schematic representation of the cross-linking. (**A**): (**a**) cross-linked and (**b**) non-cross-linked (scale bars = 5 µm). (**c**–**f**) Cumulative release over 7 days: (**c**) cumulative release over time at 12 or 48 h cross-linked for the same bioactive materials (ketoprofen); (**d**) cumulative release over time at 48 h cross-linked for different bioactive materials (MET: metformin, anticancer drug: 5-fluorouracil, macromolecule: exosomes); (**B**): (**e**) cumulative release over time for the same bioactive materials at different cross-linking time; (**f**) cumulative release over time for the same bioactive materials at different combination ratio of cross-linking time. When the same bioactive material was cross-linked at different combination ratios of cross-linking time, a controllable and stable release profile could be achieved. (Data are shown as percentage of non-cross-linked/12 h cross-linked/48 h cross-linked). All the formulations have shown more than 95% of drugs being released within 7 days; each data point represents the average of 4 samples from 4 batches (n = 4; *: *p* > 0.05 between 2 groups; #: *p* > 0.05 between all groups).

The morphology of non-cross-linked and cross-linked nanomembranes is shown in Figure 6A(a,b). As shown in Figure 6A(b), the cross-linked nanofibers kept their smooth surface, porous structures, and interconnectivity, but there were some changes in the porosity. In the present study of dissolution profiles, as shown in Figure 6A(c,d),B(e,f), all the formulations have shown more than 95% of drugs being released within 7 days in 0.05 M phosphate buffer pH 7.4. From the observations, the non-cross-linked medication achieved a drug release of more than 98% within 24 h, whereas the 48 h cross-linked sample was only able to achieve 20% drug release within 72 h (Figure 6A(c)). From the dissolution studies, all samples showed a higher percentage of drug release as dissolution time increased, achieving more than 95% by day 7 (Figure 6A(c)). In comparison of all the formulations, although individual variations existed, smaller molecules usually dissolved faster than macromolecules (Figure 6A(d)). When the same bioactive material was cross-linked at different combinations of cross-linking time, a controllable and stable release profile could be achieved (Figure 6B(e,f)). As shown, the nanomembrane is proven to be a suitable dissolution scaffold with an acceptable discriminating power.

## 4. Discussion

Chemotherapy remains a cornerstone of cancer treatment, aimed at controlling tumor progression and prolonging patient survival. However, systemic chemotherapy is often accompanied by limited efficacy and severe side effects, largely due to non-specific drug distribution to healthy tissues [30]. Adverse drug reactions can result in hospitalization, dose reductions, treatment delays, or discontinuation—ultimately compromising patient outcomes and increasing healthcare costs [31,32]. Recent advancements in melanoma treatment, particularly the use of immune checkpoint inhibitors (ICIs) and targeted therapies, have improved overall survival. However, factors such as metastasis, genetic mutations, and prior therapies significantly influence treatment decisions [33,34]. These challenges underscore the need for alternative drug delivery systems that enhance therapeutic efficacy while minimizing systemic side effects.

Postoperative recurrence following malignant tumor resection remains a significant concern. Localized drug delivery during surgical excision may reduce the risk of recurrence and systemic metastasis. Cisplatin, widely used for solid tumors including sarcomas, has demonstrated potential for intraoperative application at low doses to reduce local recurrence [35]. This study explored the localized delivery of cisplatin via electrospun gelatin nanofibers, aiming to decrease the required dosage while maintaining therapeutic efficacy. The nanofibrous membranes, fabricated via electrospinning, offer high porosity and surface area-to-volume ratio—properties that support controlled, site-specific drug release.

Cytotoxicity evaluation is critical for determining the clinical feasibility of anticancer therapies. In this study, the CCK-8 assay—a widely accepted method for assessing cellular metabolic activity—was used to quantify the cytotoxic effects of cisplatin [36]. The goal was to achieve biocompatible nanofibers within the 50–500 nm range that deliver cisplatin effectively to cancer cells at lower dosages. Experimental results confirmed the fabrication of such fibers, validated through cell viability assays and microscopy.

Cisplatin induces cytotoxicity through mechanisms such as DNA cross-linking and reactive oxygen species (ROS) generation, selectively targeting cancer cells due to their rapid proliferation and higher oxidative stress [12]. However, initial cytotoxicity results did not show heightened sensitivity in B16-F10 melanoma cells, possibly due to efflux pump mechanisms that prevent intracellular drug accumulation [37].

When cisplatin was embedded within nanofibrous gelatin membranes, its cytotoxic effects on melanoma cells increased significantly, while normal fibroblasts and keratinocytes showed minimal damage [38]. This enhancement is likely due to the nanomembrane’s ability to maintain a high local drug concentration and promote cellular uptake before efflux mechanisms can act [39].

Gelatin-based nanofibers are gaining attention in drug delivery systems (DDS) due to their excellent biocompatibility, tunable degradation rates, and ease of chemical modification [40]. Prior studies, including those by Tseng et al. and Zong et al., have demonstrated the efficacy of electrospun nanofibers loaded with cisplatin or other chemotherapeutic agents in localized cancer treatment models, with enhanced drug retention and minimal systemic toxicity [21,22]. Our previous study showed that gelatin was selected as the polymer base due to its biocompatibility and its widespread use as a wound dressing following surgery [18]. The results from this study align with these findings and further confirm the potential of electrospun gelatin nanofibers as a viable local drug delivery platform for melanoma therapy.

Recent advancements in nanotechnology-based drug delivery systems aim to overcome challenges such as bacterial resistance and the limited efficacy of traditional cancer therapies. Conventional systemic administration of chemo- and immunotherapeutics often leads to off-target effects and unpredictable outcomes. Nanomedicine offers promising solutions through targeted, multimodal delivery platforms that improve pharmacokinetics, pharmacodynamics, and tumor localization of therapeutic agents [41,42,43].

Gelatin has emerged as a valuable biomaterial in drug delivery due to its biocompatibility and functional versatility. As an energy-absorbing layer in laser bioprinting, it improves cell viability and reduces DNA damage [44]. Gelatin hydrolysate (GH) exhibits antioxidant and immunomodulatory properties and inhibits cancer cell proliferation [45]. Gelatin-based nanofibers enhance cellular uptake via endocytosis due to their nanoscale size and surface charge. Encapsulation of cisplatin in these nanofibers can bypass efflux mechanisms and increase intracellular drug accumulation, improving therapeutic efficacy [46,47].

Melanoma, a highly aggressive and treatment-resistant cancer, benefits from local drug delivery approaches that minimize systemic toxicity. Due to its high mutational burden, melanoma presents numerous neoantigens, allowing immune system recognition [48]. Our study showed that local delivery via electrospun gelatin nanomembranes is more effective than systemic administration in inducing cytotoxic effects, requiring lower drug doses (Table 2). Prior studies using cisplatin-loaded electrospun nanofibers for cervical cancer demonstrated strong mucoadhesive properties, prolonged retention, and improved therapeutic outcomes compared to intravenous delivery [22,49].

Ketoprofen was selected as a model compound for drug release characterization due to practical analytical constraints associated with direct quantification of cisplatin using UV–Vis spectroscopy. Cisplatin lacks a strong chromophore, making its detection in complex biological matrices such as gelatin challenging without advanced instrumentation. In contrast, ketoprofen exhibits a strong UV absorbance peak at ~260 nm, allowing for straightforward, reproducible quantification of release kinetics using standard UV–Vis techniques. This substitution has clear limitations. Cisplatin’s chemical behavior—including potential interactions with functional groups in the gelatin matrix (e.g., amines, thiols), aquation dynamics, and ionic state—can significantly influence its release profile in ways that ketoprofen does not replicate. Additionally, the hydrophobicity of ketoprofen may lead to an underestimation of matrix–drug interactions relevant to cisplatin. Therefore, while ketoprofen serves as a practical proxy for initial kinetic modeling and formulation optimization, it should not replace direct cisplatin release studies for final translational assessment [50].

This study has several limitations: (1) The murine B16-F10 melanoma cell line was selected for its well-established use in preclinical melanoma research; the absence of a human melanoma cell line, such as A375, limits the direct translational relevance to human disease. (2) Cytotoxic effects were assessed morphologically; quantitative assays are needed for confirmation. (3) This study used in vitro models; future research will involve 3D bioprinted skin and hydrogel-based tissue mimics [51], followed by animal models and, eventually, clinical trials. (4) Further work is needed to optimize targeted delivery, enhance scaffold biocompatibility, test with different chemotherapeutics, and explore applications in other cancers.

Nanoparticles provide targeted, sustained drug release while minimizing systemic side effects. Despite challenges in maintaining nanocarrier stability in circulation [52], advances in design allow effective drug delivery and tumor-specific accumulation. Neoadjuvant chemotherapy improves survival but can impair recovery post-surgery [53]; local nanocarrier delivery during surgery offers a strategy to improve therapeutic outcomes with fewer adverse effects.

## 5. Conclusions

Embedding cisplatin into gelatin nanomembranes for local application not only reduces systemic toxicity and total drug usage but also lowers production costs. These nanomembranes may also be used for tumor vessel embolization, offering a less invasive alternative to open surgery. Controlled cross-linking and degradation rates can enable stable, time-regulated cisplatin release at therapeutic levels without systemic toxicity.

## Figures and Tables

**Table 1:** Cisplatin concentration (µg/mL): 100 µg/mL sample. Specifically, 100 µg/mL: 5 mg cisplatin in 50 cc H_2_O (with 6 gm gelatin). A total of 3 gm gelatin becomes a 20 × 60 cm nanomembrane = 1200 cm^2^; 2.5 mg/1200 cm^2^ = 0.002083333 mg/cm^2^ = 2.083333 µg/cm^2^. Therefore, 2.5 mg cisplatin in 1200 cm^2^ nanomembrane = 2.083333 µg/cm^2^.

Cisplatin	Amount in 1 × 1 cm Nanomembrane	Amount in 1 mL Medium
100	2.083333 µg	100 µg/mL × amount of medium (mL)
50	1.042 µg	50 µg/mL × amount of medium (mL)
25	0.521 µg	25 µg/mL × amount of medium (mL)
12.5	0.26 µg	12.5 µg/mL × amount of medium (mL)
6.25	0.13 µg	6.25 µg/mL × amount of medium (mL)
3.125	0.065 µg	3.125 µg/mL × amount of medium (mL)

**Table 2:** Cell Viabilities of Tissue Cultures Across Varying Treatment Times and Concentrations.

Treatment Time	Cisplatin Concentration (µg/mL)						
	100	50	25	12.5	6.25	3.125	Control
L929 Fibroblasts							
24 h	14.7%	15.1%	13.7%	15.7%	39.5%	64.8%	100%
48 h	2.1%	0.3%	2.1%	3.4%	11.7%	31.0%	100%
72 h	0.7%	−0.8%	−0.7%	1.3%	7.2%	38.7%	100%
B16-F10 Melanoma Cells							
24 h	10.8%	19.2%	21.4%	33.7%	55.5%	94.5% *	100%
48 h	−0.2%	−1.7%	−1.8%	10.6%	23.8%	54.8%	100%
72 h	−0.4%	−1.5%	−1.4%	4.6%	16.4%	36.5%	100%
HaCaT Keratinocytes							
24 h	3.5%	1.8%	4.2%	9.7%	45.5%	81.0%	100%
48 h	−0.6%	−0.6%	−0.5%	0.7%	5.5%	15.9%	100%
72 h	−0.5%	−1.2%	−0.4%	0.0%	1.1%	5.4%	100%

Note: A cell viability less than or equal to 0 indicates the absence or minimal presence of living cells in the experimental group. * *p* > 0.05.

**Table 3:** Pure Gelatin Nanomembrane Biocompatibility (n = 6).

Group	Cell Viability (%)	SD (%)	*p*
100%	109.4%	18.3%	0.252
75%	121.3%	20.4%	0.031
50%	113.7%	19.6%	0.126
25%	87.5%	15.1%	0.079
Negative Control (0%)	100.0%	4.4%	1.000
Positive Control (10% DMSO)	0.0%	0.6%	0.000

## Data Availability

The datasets used and/or analyzed in this study are available from the corresponding author upon reasonable request.
